# Understanding the Interplay Between Obesity and Cancer: From Mechanisms to Therapeutic Opportunities

**DOI:** 10.3390/cancers18101620

**Published:** 2026-05-17

**Authors:** Sunsook Hwang, Byungjoo Kim, Seungyeon Yang, Seung Min Jeong

**Affiliations:** 1 Department of Immunology, School of Medicine, Konkuk University, Chungju 27478, Republic of Korea; sunhwang@kku.ac.kr (S.H.);; 2 Research Institute of Medical Science, KU Open Innovation Center, Chungju 27478, Republic of Korea; 3 Department of Biochemistry, College of Medicine, The Catholic University of Korea, Seoul 06591, Republic of Korea; 4 Institute for Aging and Metabolic Diseases, College of Medicine, The Catholic University of Korea, Seoul 06591, Republic of Korea

**Keywords:** obesity, tumor progression, therapy resistance, tumor microenvironment, inflammation, metabolic reprogramming

## Abstract

Obesity has risen to the forefront of global public health challenges, particularly due to its association with several chronic diseases. Its association with cancer has attracted significant research interest. The relationship between obesity and cancer is complex and profoundly influences tumorigenesis, cancer progression, metastasis, and therapeutic efficacy. This review provides the molecular basis of these interactions, elucidating how obesity triggers changes in the tumor microenvironment, disrupts metabolic pathways, and promotes inflammation. These factors facilitate cancer development and reduce the efficacy of treatments. By unraveling these mechanisms, we aim to identify therapeutic strategies that could mitigate obesity’s detrimental effects on cancer outcomes and contribute to the development of more effective strategies for managing obesity-related cancers.

## Simple Summary

Obesity is a growing global health problem that affects not only general health but also cancer risk and treatment outcomes. People with obesity are more likely to develop certain cancers, and their tumors often respond less effectively to therapy. This is partly because excess body fat alters the microenvironment around tumors, changes how cancer cells use energy, and alters abnormal adipose tissue-derived signals that promote tumor growth. In this review, we summarize how these obesity-related changes contribute to cancer progression and treatment resistance, and we discuss potential strategies to improve therapy. A better understanding of these mechanisms may support the development of more effective treatments for patients with obesity-related cancers.

## 1. Introduction

Obesity is defined by the World Health Organization (WHO) as abnormal or excessive fat accumulation that presents a risk to health, conventionally assessed by a body mass index (BMI) ≥ 30 kg/m^2^. This definition emphasizes excessive adipose tissue accumulation—particularly visceral fat—as the central pathophysiological feature linking obesity to its downstream metabolic and inflammatory consequences, which are discussed in subsequent sections.

Conceptually, obesity contributes to cancer through many of the established hallmarks of cancer [1,2]. The hallmarks most strongly affected by obesity include sustaining proliferative signaling and resisting cell death, driven by chronic activation of insulin/IGF-1 and adipokine-mediated pathways; deregulating cellular energetics, through systemic and local metabolic reprogramming; tumor-promoting inflammation, mediated by adipose tissue-derived cytokines; genome instability, due to oxidative stress and impaired DNA repair; and avoiding immune destruction, through obesity-driven immunosuppression. This hallmark-based framework provides a useful conceptual structure for the mechanistic sections that follow.

A particularly relevant concept that integrates these mechanisms is metaflammation—the chronic, low-grade, metabolically driven inflammation characteristic of obesity [3,4,5]. Unlike acute inflammation, metaflammation arises from the convergence of metabolic dysregulation and immune activation in expanded adipose tissue, and it serves as a conceptual bridge between obesity and the tumor-promoting inflammation discussed throughout this review.

Obesity is a global health crisis, with its prevalence having more than tripled since 1975. According to recent data from the World Health Organization (WHO), approximately 43% of adults (aged 18 and older) worldwide were overweight, and 16% were obese in 2022 [6]. The prevalence of obesity among children and adolescents has also increased dramatically. In 2022, 160 million children aged 5–19 years were living with obesity. Excessive body fat significantly impacts an individual’s overall health and well-being, contributing to chronic diseases such as cardiovascular disease, type 2 diabetes, and various cancers [7,8].

Among the various chronic diseases associated with obesity, cancer stands out because of its particularly strong association. Obesity is not only associated with a higher risk of developing cancer, but also with increased rates of cancer recurrence and mortality among cancer survivors [9,10,11]. The complex interplay between obesity and cancer involves changes in several cellular and molecular pathways [12,13]. Obesity creates a pro-inflammatory state characterized by dysregulated adipokine secretion, chronic low-grade inflammation and alterations in metabolic pathways, all of which contribute to tumorigenesis and cancer progression [14,15,16].

Despite advances in cancer therapies, the efficacy of treatments such as immunotherapy, chemotherapy and targeted therapies is often reduced in patients with obesity [17,18]. The immunosuppressive tumor microenvironment (TME) in obesity, characterized by the recruitment of regulatory T cells and myeloid-derived suppressor cells (MDSCs), reduces the efficacy of immune-based treatments [19]. In addition, chronic inflammation and metabolic dysregulation impair responses to major cancer treatments, including chemotherapy, immunotherapy, and radiotherapy, highlighting the need for novel therapeutic strategies that specifically address the unique challenges caused by obesity [20].

Here, we review the multifaceted relationship between obesity and cancer, focusing on its impact on tumorigenesis, cancer progression, metastasis and resistance across multiple anticancer treatment modalities. This review aims to explore the potential of modulating the TME, inflammation, and metabolic pathways to improve the efficacy of cancer treatment in patients with obesity. By targeting these key areas, current and emerging approaches may help overcome the barriers posed by obesity in cancer therapy. Achieving this goal will require a concerted approach that integrates clinical, translational and basic research to develop and implement more effective and personalized treatment strategies for obese cancer patients.

## 2. Methodology

This narrative review was conducted using a structured literature search to identify peer-reviewed studies examining the interplay between obesity and cancer. PubMed/MEDLINE, Scopus, and Web of Science were searched, with the final search updated in May 2026. Search terms included combinations of “obesity,” “adiposity,” “BMI,” or “body mass index” with “cancer,” “tumor,” “tumorigenesis,” “metastasis,” “therapy resistance,” “chemotherapy,” “immunotherapy,” “radiotherapy,” “tumor microenvironment,” “adipokine,” “inflammation,” “metabolic reprogramming,” “weight loss,” “GLP-1,” and “bariatric surgery.” The primary search was restricted to articles published between January 2000 and May 2026, with selective inclusion of seminal studies where relevant. Eligible studies included peer-reviewed original research articles, systematic reviews, and meta-analyses published in English, including both preclinical and clinical studies. Where available, obesity-stratified or obesity-specific evidence was prioritized. Non-English articles, conference abstracts without full data, opinion pieces without primary data, and studies not directly addressing obesity–cancer relationships were excluded. Article selection was conducted independently by two authors, with disagreements resolved by consensus. Clinical evidence was prioritized over preclinical evidence when both were available, whereas preclinical studies were retained when they provided mechanistic insight.

## 3. Obesity in Tumorigenesis, Progression, and Metastasis

### 3.1. Obesity and Tumorigenesis

At the initiation stage of carcinogenesis, obesity contributes to the acquisition of driver mutations through several converging mechanisms. Chronic oxidative stress and elevated reactive oxygen species (ROS) production in expanded adipose tissue cause direct DNA damage in adjacent and circulating cells, while concurrent low-grade inflammation impairs the activity of key DNA repair enzymes [1,2,3]. In addition, obesity-driven hyperinsulinemia, elevated IGF-1, and adipose-derived hormonal changes increase the likelihood of spontaneous replication errors during cellular proliferation [14,15]. Together with the promotion and progression mechanisms described below, these initiation-stage processes help explain how obesity facilitates carcinogenesis.

**Figure 1:**
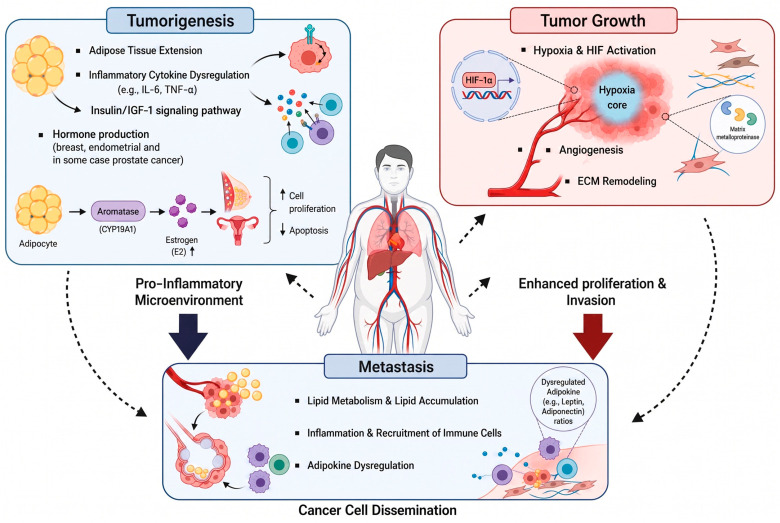
Obesity-driven mechanisms promoting tumorigenesis, progression and metastasis. Obesity contributes to cancer development and progression through multiple interconnected mechanisms. During tumorigenesis, adipose tissue expansion induces a pro-inflammatory microenvironment characterized by cytokine dysregulation (e.g., IL-6, TNF-α) and activation of insulin/IGF-1 signaling pathways, which promote cellular transformation and proliferation. In tumor growth, obesity-associated hypoxia leads to HIF-1α activation and drives aberrant angiogenesis and extracellular matrix (ECM) remodeling, resulting in enhanced tumor cell proliferation and invasion. As the disease progresses, these changes collectively facilitate metastasis. In metastatic stages, obesity promotes lipid metabolism and accumulation within tumor cells, supports inflammatory signaling and immune cell recruitment, and disrupts adipokine balance (e.g., leptin, adiponectin), thereby enhancing cancer cell survival, dissemination, and colonization of distant organs. Together, these processes establish a tumor-promoting environment that accelerates cancer progression and metastatic spread.

Obesity is linked to an increased risk of developing at least 13 different types of cancer, including colorectal, breast, endometrial, and kidney cancers [21]. This association is primarily due to the pro-inflammatory state induced by obesity, characterized by elevated levels of adipokines, cytokines, and insulin, which collectively create a favorable milieu for tumor initiation and progression [22] (Figure 1). For example, inflammation within adipose tissue leads to the release of pro-inflammatory adipocytokines, such as interleukin-6 (IL-6) and tumor necrosis factor-alpha (TNF-α) [23,24]. These cytokines are known to play a pivotal role in the development of colorectal cancer by promoting an environment that favors cellular transformation and proliferation. Moreover, obesity increases levels of circulating insulin and subsequently activates the insulin-like growth factor 1 (IGF-1) signaling pathway, leading to insulin resistance [25]. This pathway is critical in the development of cancer, as it promotes cell proliferation and inhibits apoptosis, offering a direct mechanism through which obesity can facilitate cancer progression [26]. The activation of the insulin/IGF-1 pathway not only encourages the growth of existing tumor cells but may also play a role in the initiation of new cancerous growths by affecting cellular DNA and promoting mutations [27].

Additionally, the role of adipose tissue extends beyond the production of inflammatory mediators. Adipose tissue-derived stem cells and immune cells infiltrate the tumor microenvironment, further facilitating tumorigenesis by promoting angiogenesis and suppressing antitumor immune responses [16]. Furthermore, increased estrogen production observed in obesity, especially relevant in breast and endometrial cancers, represent another important mechanism [28]. Adipose tissue, particularly in postmenopausal women, serves as a significant site of estrogen synthesis. Elevated levels of estrogen have been linked to increased cell proliferation and reduced apoptosis in estrogen-sensitive tissues, thus elevating the risk of female-related cancers [29,30].

### 3.2. Obesity and Cancer Progression

Obesity-induced alterations in the tumor microenvironment play a pivotal role in cancer progression (Figure 1). The expansion of adipose tissue in obese individuals promotes a series of pathophysiological changes, including hypoxia, angiogenesis, and remodeling of the extracellular matrix (ECM), thereby facilitating tumor growth and invasion [16,31,32]. While intratumoral hypoxia is a general feature of solid tumors, emerging evidence suggests that obesity may enhance hypoxia-associated signaling and its downstream consequences. Although direct intratumoral oxygen tension measurements in patients with and without obesity remain limited, this relationship is supported by preclinical obese mouse models [16,32].

Firstly, obesity-induced hypoxia in the tumor microenvironment significantly promotes cancer growth and progression through several key mechanisms. Hypoxia triggers the activation of hypoxia-inducible factors (HIFs), which in turn upregulate genes involved in angiogenesis, metabolism, and cell survival [33,34]. This leads to increased formation of new blood vessels to supply the tumor with oxygen and nutrients, although these vessels are often dysfunctional, contributing to uneven blood flow and persistent hypoxic regions [35,36]. Furthermore, hypoxia drives cancer cells to rely more on glycolysis for energy, supporting their growth in low-oxygen conditions and producing byproducts that stimulate tumor progression. Additionally, hypoxia facilitates cancer cell invasion and metastasis by promoting epithelial–mesenchymal transition (EMT), thereby enhancing the ability of cancer cells to spread [34].

Next, obesity significantly impacts the tumor microenvironment by enhancing the secretion of pro-angiogenic factors such as vascular endothelial growth factor (VEGF) and angiopoietin-2, which are crucial for the development of new blood vessels within tumors [37,38]. This angiogenesis is key to providing tumors with the necessary nutrients and oxygen to sustain their growth and spread. Furthermore, obesity leads to the dysregulated secretion of adipokines, such as leptin, adiponectin, and interleukin-6 (IL-6), which play a significant role in cancer progression. These adipokines contribute to tumorigenesis by promoting cancer cell proliferation, survival, and invasion through the activation of critical signaling pathways, including PI3K/Akt and MAPK/ERK [39,40,41].

In addition, matrix metalloproteinases (MMPs), particularly MMP-2 and MMP-9, are upregulated in obese adipose tissue and the tumor microenvironment. MMPs degrade basement membrane and stromal collagen components, releasing sequestered growth factors and facilitating tumor cell invasion across tissue boundaries [13,16]. Obesity-driven inflammatory cytokines (e.g., IL-6, TNF-α) and adipokines further enhance MMP expression in stromal and tumor cells, providing a mechanistic link between adipose tissue dysfunction and the ECM-remodeling events described below [23,25].

Finally, obesity-induced alterations in the ECM significantly contribute to cancer growth and metastasis by increasing matrix stiffness, which in turn promotes cancer cell proliferation and invasion [42]. These changes enhance signaling pathways such as integrin signaling, which is crucial for tumor aggressiveness. Additionally, the remodeled ECM in obesity modulates the availability of growth factors, thereby facilitating tumor progression through enhanced cell proliferation and angiogenesis [43]. Moreover, the altered ECM composition and structure facilitate cancer cell detachment, motility, and invasion, promoting metastasis [44].

### 3.3. Obesity and Metastasis

Metastasis, in which cancer cells disseminate from the primary tumor site to distant organs, is a pivotal step in cancer progression and is often indicative of poor clinical outcomes [45]. This process is significantly influenced by obesity, as evidenced by findings indicating that patients with obesity and metastatic melanoma exhibit shorter progression-free and overall survival compared to their non-obese counterparts [46]. Furthermore, obesity is linked to an increased incidence of metastasis to critical organs such as the liver and lung, underscoring its detrimental role in cancer dissemination [47].

The mechanisms through which obesity fosters metastasis are multifaceted and primarily involve alterations in the TME [48]. One key mechanism is the priming of premetastatic niches. Obesity-associated inflammation and stromal activation can remodel distant tissues, such as the lung, even prior to tumor cell arrival, thereby enhancing the recruitment of myeloid populations and establishing a permissive environment for metastatic colonization. This is consistent with the broader concept of premetastatic niche formation, in which inflammatory cytokines, extracellular matrix remodeling, and myeloid cell trafficking collectively create favorable landing sites for circulating tumor cells.

In addition, adipose-rich environments provide metabolic support that facilitates metastatic growth. Adipocytes supply abundant lipids, which are efficiently utilized by tumor cells. CD36—a fatty acid translocase—is markedly upregulated in metastatic cancer cells [49,50]. Under obesity-associated lipid-rich conditions, CD36 promotes metastasis through multiple mechanisms, including (i) sustaining energy production via β-oxidation, (ii) facilitating membrane remodeling and activating pro-migratory signaling pathways such as Src–Akt–ERK and Rac1-mediated cytoskeletal reorganization, and (iii) buffering lipotoxic stress through lipid storage, thereby enhancing tumor cell survival during dissemination [49,51,52,53].

Finally, obesity-induced chronic inflammation further amplifies metastatic potential by recruiting tumor-associated macrophages and other pro-tumorigenic immune populations, which promote tumor cell invasion and support niche formation in distant organs [47,54,55] (Figure 1).

## 4. Obesity and Therapy Resistance

### 4.1. Tumor Microenvironment Remodeling

Obesity plays a significant role in inducing therapy resistance in cancer by intricately altering the TME [16,56]. The structural and functional changes triggered by obesity create conditions that not only support cancer cell growth and survival but also hinder the effectiveness of diverse anticancer strategies [57,58].

**Figure 2:**
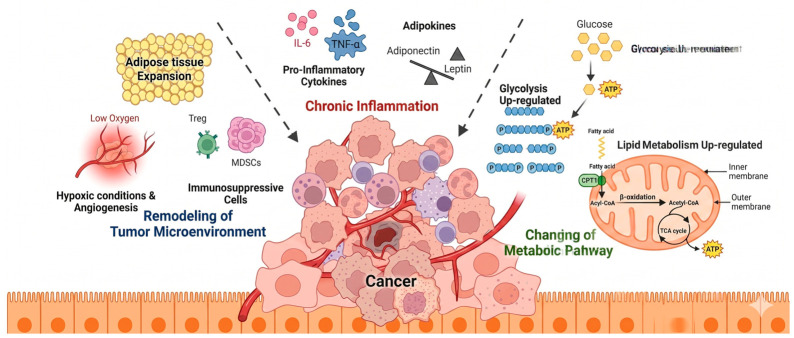
Obesity-driven tumor microenvironment remodeling, chronic inflammation, and metabolic reprogramming contributing to chemotherapy resistance. Adipose tissue expansion induces hypoxia and aberrant angiogenesis, leading to poorly organized vasculature that limits drug delivery, while extracellular matrix remodeling and the accumulation of immunosuppressive cells impair anti-tumor immunity. Concurrently, chronic inflammation—characterized by elevated pro-inflammatory cytokines (e.g., IL-6, TNF-α) and adipokine imbalance, including increased leptin and reduced adiponectin—activates oncogenic signaling pathways that enhance tumor cell survival and adaptation to therapeutic stress. In parallel, metabolic reprogramming, including increased glycolysis and lipid metabolism, supports energy production and promotes resistance to treatment-induced cell death. Collectively, these interconnected processes establish a tumor-promoting niche that reduces the efficacy of chemotherapy, targeted therapy, and immunotherapy.

The expansion of adipose tissue, a characteristic feature of obesity, leads to a series of detrimental modifications within the TME including hypoxia, abnormal angiogenesis, and extracellular matrix remodeling (Figure 2). As adipose tissue expands, vascular supply becomes insufficient, leading to local hypoxia, HIF-1α activation, and a dysfunctional angiogenic response characterized by increased VEGF and related mediators [59]. Rather than restoring normal perfusion, this response often produces aberrant, poorly organized vasculature, thereby sustaining metabolic stress and limiting the effective intratumoral distribution of systemic therapies. In parallel, obesity promotes extracellular matrix remodeling and desmoplasia, with increased collagen-rich fibrosis and tissue stiffening that reinforce pro-survival signaling, alter cell–matrix interactions, and further restrict drug penetration [60]. These conditions collectively form a supportive niche that limits the effects of therapeutic agents.

Importantly, the leptin-to-adiponectin ratio—rather than the absolute concentration of either adipokine alone—is increasingly recognized as the most informative biomarker of obesity-driven adipose dysfunction in oncologic contexts. Moreover, a defining and integrative feature of the obese TME is adipokine imbalance. Elevated leptin and reduced adiponectin levels shift signaling networks toward a pro-tumorigenic state [61,62]. Leptin activates pathways such as JAK/STAT, PI3K/AKT, MAPK, and VEGF signaling, promoting tumor cell proliferation, angiogenesis, and adaptive responses to therapeutic stress [63,64]. In contrast, reduced adiponectin diminishes AMPK-mediated inhibitory signaling, removing constraints on tumor growth and vascular remodeling [62,65]. Additional adipokines, including resistin and visfatin, may further reinforce these effects in a tumor-type-specific manner [39,66,67].

### 4.2. Induction of Chronic Inflammation

Obesity contributes to cytokine-based cancer therapy resistance through the induction of chronic inflammation, which involves a complex interplay of immune cell alterations, cytokine production, and intracellular signaling pathways [68]. Characterized by chronic low-grade inflammation, obesity leads to the infiltration of immune cells into adipose tissue, which, along with adipocytes, secrete pro-inflammatory cytokines such as TNF-α, IL-6, and IL-1β (Figure 2). These cytokines not only foster an inflammatory tumor microenvironment but also activate signaling pathways that directly contribute to therapy resistance [69]. For instance, the activation of JAK/STAT pathway by IL-6 results in the transcriptional upregulation of anti-apoptotic genes, including Bcl-2 and Bcl-xL, enhancing cancer cell survival and rendering them resistant to apoptosis [70]. Similarly, TNF-α-mediated activation of the NF-κB signaling pathway induces the expression of genes that further promote cell survival and inflammation [71]. Moreover, these cytokines can activate intracellular pathways that increase the expression of drug efflux transporters, such as P-glycoprotein [72,73]. The elevated expression of these transporters decreases the intracellular concentration of chemotherapy drugs, thereby reducing their effectiveness and contributing to chemotherapy resistance.

Beyond affecting cancer cell survival directly, obesity-induced inflammation impairs the function of various immune cells crucial for tumor surveillance and antitumor responses [74]. Chronic exposure to pro-inflammatory cytokines leads to T cell dysfunction and exhaustion, reduced cytotoxicity of NK cells, and macrophage polarization towards a phenotype that supports tumor growth [75,76,77]. In addition, obesity-induced inflammation favors the recruitment and activity of immunosuppressive cells, such as regulatory T cells (Tregs) and myeloid-derived suppressor cells (MDSCs) [78]. These cells dampen the immune response and promote an environment of immune tolerance towards cancer cells, further enhancing therapy resistance [75].

These changes not only facilitate immune evasion by cancer cells but also undermine the efficacy of immunotherapies that depend on a robust immune response to target tumors effectively.

### 4.3. Modulation of Metabolism Pathways

Obesity’s impact on cancer therapy resistance extends to profound metabolic dysregulation within cancer cells, a consequence of obesity’s complex interplay with insulin resistance, dyslipidemia, and altered glucose metabolism (Figure 2). These metabolic disturbances give rise to a tumor environment that can reduce chemotherapeutic efficacy by supporting cancer cell survival and limiting antitumor immune response [79,80]. For example, dysregulated lipid metabolism and heightened fatty acid synthesis observed in obesity can contribute to chemotherapy resistance by bolstering cancer cell survival and undermining the effectiveness of drug-induced apoptosis [81]. Recently, it has been shown that chemotherapy induces fatty acid oxidation in cancer cells, which is required for proper cell death after DNA damage. However, this metabolic response is thwarted in the context of obesity, leading to an impairment in Casp2 (caspase-2) acetylation and, consequently, a reduction in chemotherapy efficacy, as demonstrated in obese mouse models [82].

Obesity-induced metabolic alterations also exacerbate competition within the tumor microenvironment, creating a scarcity of nutrients and energy substrates that adversely affects immune cell function [13,83]. Cancer cells, by outcompeting immune cells for vital resources such as glucose, can impair immune surveillance and response, further diminishing the effectiveness of immunotherapies [84,85]. Additionally, the changes in adipokine secretion and the inflammatory milieu associated with obesity can alter metabolic pathways and signaling cascades critical for immune cell activation and function, thereby impacting the response to immunotherapies [22,86].

## 5. Therapeutic Strategies to Overcome Obesity-Driven Therapy Resistance

Understanding the intricate relationship between obesity and cancer therapy resistance has paved the way for the development of novel therapeutic strategies aimed at modulating the TME, inflammation, or metabolism to improve antitumor immunity and therapeutic responses [58,87]. Here, we discuss several promising approaches that target the unique challenges posed by obesity in cancer therapy.

### 5.1. Targeting the Tumor Microenvironment

**Figure 3:**
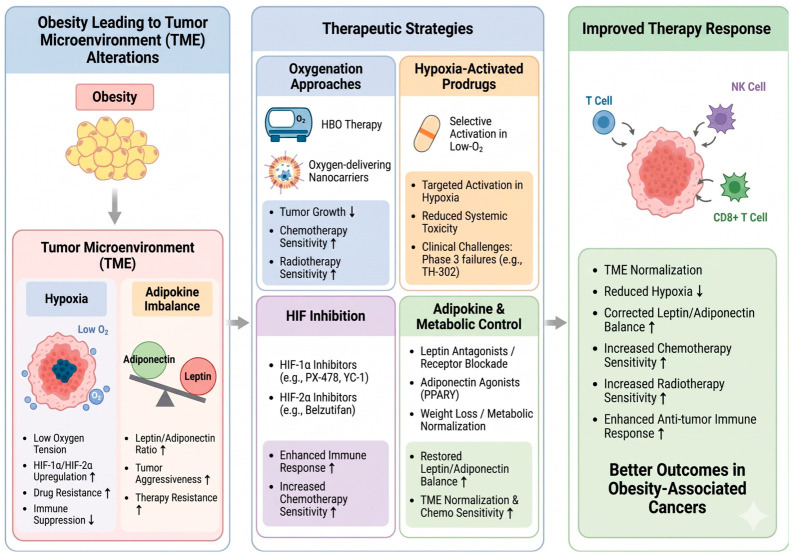
Therapeutic strategies targeting obesity-driven tumor microenvironment remodeling to improve treatment response. Therapeutic approaches aimed at normalizing the tumor microenvironment enhance treatment efficacy. Oxygenation strategies, including hyperbaric oxygen therapy and oxygen-delivering nanocarriers, are used to alleviate hypoxia and improve sensitivity to chemotherapy and radiotherapy. Hypoxia-activated prodrugs selectively target low-oxygen tumor regions, increasing therapeutic specificity while minimizing systemic toxicity. In parallel, inhibition of HIF signaling disrupts hypoxia-driven adaptive responses that support tumor survival under treatment stress. Additionally, modulation of adipokine and metabolic pathways—such as leptin blockade, adiponectin restoration, and metabolic interventions—aims to rebalance tumor-promoting signals and improve treatment responsiveness. These strategies promote tumor microenvironment normalization, enhance drug delivery and immune function, and ultimately improve the efficacy of anticancer therapies.

Targeting the TME represents a critical strategy for overcoming therapy resistance in obesity (Figure 3). In particular, modulation of tumor hypoxia has emerged as a key approach to enhance the efficacy of conventional therapies. Increasing tumor oxygenation can improve drug delivery, sensitize cancer cells to radiotherapy and cytotoxic agents, and promote antitumor immune responses.

Preclinical studies provide evidence supporting this strategy. For example, hyperbaric oxygen (HBO) therapy alleviates hypoxia, reduces HIF-1α signaling, and mitigates drug resistance, thereby enhancing the efficacy of chemotherapy [88]. Notably, the combination of HBO with liposomal doxorubicin (Doxil) demonstrated synergistic tumor growth inhibition (~91%) without additional toxicity, primarily through improved drug penetration and chemosensitization. However, as a systemic oxygenation strategy, HBO presents logistical challenges and potential safety concerns, which have limited its adoption in routine clinical oncology. To address these limitations, localized approaches such as oxygen-delivering nanocarriers have been developed. These systems can deliver oxygen and chemotherapeutic agents simultaneously, alleviating hypoxia and enhancing drug efficacy. For instance, oxygen-loaded doxorubicin nanocarriers increased apoptosis markers under hypoxic conditions compared with free drug in vitro, supporting their potential to overcome hypoxia-driven chemoresistance [89].

Alternatively, hypoxia can be therapeutically exploited using hypoxia-activated prodrugs (HAPs), which are selectively activated in low-oxygen environments to target hypoxic tumor regions. Although several HAPs have demonstrated promising activity in early-phase clinical trials, some agents (e.g., TH-302/evofosfamide) failed in phase 3 studies [90]. These outcomes highlight that hypoxia-targeting strategies require validated biomarkers, rational combination regimens, and appropriate patient selection, rather than uniform application across tumor types or patient populations [91,92,93]. Key limitations include the lack of prospectively applied hypoxia biomarkers (e.g., pimonidazole, ^18^F-FMISO PET) for patient enrichment, off-target reductive metabolism in normoxic tissues that limits achievable dose, and the absence of obesity-stratified efficacy data despite the biological rationale that obesity-amplified intratumoral hypoxia should enhance HAP activation. Future HAP trials in obesity-associated cancers should therefore incorporate hypoxia biomarker confirmation with BMI and body-composition stratification [91,92,93].

At the molecular level, hypoxia-inducible factors (HIF-1/2) function as central regulators of hypoxia-driven therapy resistance, making them attractive therapeutic targets. Clinically, HIF-2α inhibition has been validated by the approval of belzutifan for von Hippel–Lindau (VHL)-associated tumors [94]. Although HIF-1α inhibition is currently supported primarily by preclinical and early-phase studies, it remains an active area of investigation. For example, PX-478 reduces tumor hypoxia and enhances chemotherapy efficacy; in pancreatic ductal adenocarcinoma models, its combination with gemcitabine increased tumor-infiltrating T cells and improved anti-tumor responses [95]. Similarly, YC-1 enhances the efficacy of gefitinib in non-small-cell lung cancer by promoting EGFR degradation, thereby overcoming resistance [96]. However, it is important to note that direct experimental evidence demonstrating obesity-specific efficacy of HIF-1α inhibition remains lacking.

Beyond hypoxia, adipokine dysregulation represents another key TME feature in obesity. Strategies targeting leptin signaling (e.g., leptin antagonists or receptor blockade), increasing adiponectin (e.g., via PPARγ agonists), or correcting systemic metabolic imbalance through weight loss may normalize the tumor microenvironment [97,98]. Such interventions are expected to reduce tumor aggressiveness and improve therapeutic sensitivity. Nevertheless, direct clinical evidence supporting adipokine-targeted therapies in obese cancer patients remains limited. Currently, the most evidence-based approaches involve weight loss and metabolic control, which have been shown to decrease leptin levels, increase the adiponectin-to-leptin ratio, and correlate with improved cancer-related outcomes in breast cancer survivors [99].

Overall, TME remodeling strategies are supported by strong mechanistic rationale and robust preclinical evidence, although obesity-stratified clinical validation remains limited.

### 5.2. Targeting Chronic Inflammation

**Figure 4:**
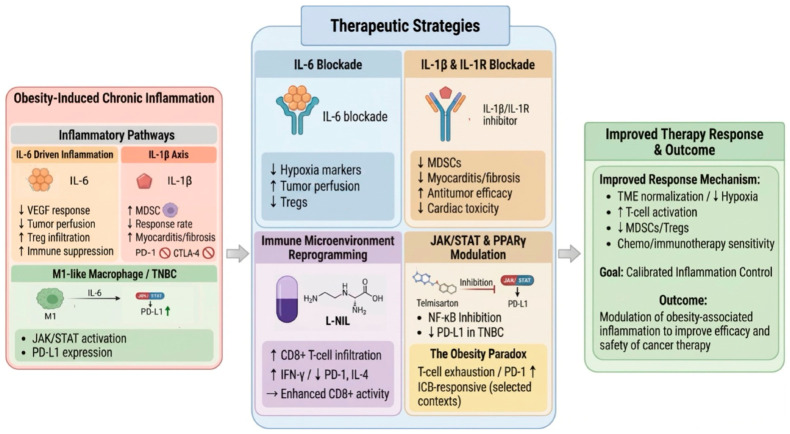
Therapeutic strategies targeting obesity-associated chronic inflammation to improve cancer treatment efficacy. This schematic highlights therapeutic approaches designed to modulate obesity-associated inflammatory pathways and enhance cancer treatment outcomes. Targeted inhibition of key inflammatory axes, including IL-6 and IL-1β/IL-1 receptor signaling, can improve tumor perfusion, reduce immunosuppressive cell populations such as myeloid-derived suppressor cells, and enhance antitumor efficacy. Immune microenvironment reprogramming strategies, including inhibition of iNOS (e.g., L-NIL), promote CD8^+^ T cell infiltration and effector function. In addition, modulation of intracellular signaling pathways such as JAK/STAT and PPARγ can reduce PD-L1 expression and restore immune balance. These approaches aim to recalibrate, rather than completely suppress, inflammation, thereby improving the effectiveness and safety of chemotherapy and immunotherapy. Overall, targeted control of inflammation represents a promising strategy to enhance therapeutic response in obesity-associated cancers.

Another important strategy for improving cancer treatment outcomes in obesity is to target obesity-associated inflammation (Figure 4). Among the inflammatory mediators implicated in therapy resistance, IL-6 has emerged as one of the most consistent candidates. In obese breast cancer models, obesity-induced inflammation impaired responsiveness to anti-VEGF therapy, and IL-6 inhibition specifically reversed these resistance mechanisms [37]. IL-6 blockade improved tumor perfusion, reduced hypoxia-marker expression, and attenuated anti-VEGF-induced infiltration of CD4^+^CD25^+^ regulatory T cells in obese tumors. Notably, combining IL-6 inhibition with anti-VEGF therapy and doxorubicin significantly suppressed tumor growth in obese tumor-bearing mice, with minimal additional benefit in lean animals, supporting an obesity-conditioned therapeutic window and a mechanistic link between IL-6-driven inflammation, immunosuppression, and reduced treatment efficacy [37]. However, this evidence remains preclinical: although IL-6 pathway-blocking agents (e.g., siltuximab, tocilizumab) are clinically available for non-oncologic indications, obesity-stratified randomized trials of therapeutic IL-6 blockade as a cancer-treatment adjuvant have not yet been conducted.

Interleukin-1β (IL-1β)-driven inflammation is another obesity-linked axis with strong causal evidence across tumor models. In renal cancer, obesity was associated with worse clinical outcomes with standard of care anti-programmed cell death protein 1 (PD-1) therapy, and in obesity mouse models, elevated IL-1β and MDSC accumulation were linked to reduced response rates; neutralizing IL-1β in obese mice improved response frequency and reduced intratumoral MDSCs [17]. In pancreatic ductal adenocarcinoma (PDAC), an orthotopic high-fat diet obesity model treated with FOLFIRINOX plus dual checkpoint blockade (anti-PD-1 + anti-CTLA-4) showed elevated IL-1β, severe cardiac immune-related adverse events, and limited therapeutic benefit; IL-1β (or IL-1R) blockade simultaneously reduced myocarditis and cardiac fibrosis and enhanced antitumor efficacy, with improved survival and a tumor microenvironment shift toward fewer Gr1^+^ suppressive myeloid cells and more CD8^+^IFN-γ^+^ effector T cells [100]. Clinically, IL-1β blockade with canakinumab—although FDA-approved for non-oncologic indications and with a generally favorable safety profile—has not been prospectively tested as a cancer-therapy adjuvant in obesity-stratified randomized trials.

Combining chemotherapy with immune-modulating strategies can reprogram the obesity-altered tumor microenvironment and improve treatment response. Specific agents such as L-NIL, an inducible nitric oxide synthase (iNOS) inhibitor, have been used to enhance antitumor immune responses by reducing immunosuppressive cell populations such as MDSCs and Tregs and increasing CD8+ T cells infiltration [101]. Obesity-related M1 macrophages secrete IL-6 through a JAK/STAT-dependent pathway to enhance PD-L1 expression in triple-negative breast cancer (TNBC). Telmisartan reverses this by activating peroxisome proliferator-activated receptor (PPAR-γ) and inhibiting NF-κB p65, highlighting its potential as an adjunctive therapy in TNBC [102].

Importantly, obesity-associated inflammation exerts context-dependent effects on immunotherapy. While chronic inflammatory signaling contributes to immunosuppression and therapy resistance, obesity-induced immune dysregulation, such as T-cell exhaustion and PD-1 upregulation, can paradoxically increase responsiveness to immune checkpoint blockade in selected settings [103,104]. Consistent with this obesity paradox, several clinical and translational studies report improved outcomes in patients with obesity treated with PD-1/PD-L1 or CTLA-4 inhibitors. However, this benefit is not unconditional: persistent systemic inflammation is also associated with higher rates of immune-related adverse events, and elevated inflammatory markers, including the systemic immune-inflammation index, IL-6, and IL-8, correlate with poorer survival in patients receiving immune checkpoint inhibitors [105,106,107]. Taken together, these observations suggest that inflammation in obesity functions as a modifiable, rather than uniformly detrimental, determinant of immunotherapy response. Accordingly, strategies that recalibrate, rather than fully suppress, obesity-associated inflammation may maximize the therapeutic benefit of immune checkpoint blockade in this population.

### 5.3. Targeting Metabolic Pathways

**Figure 5:**
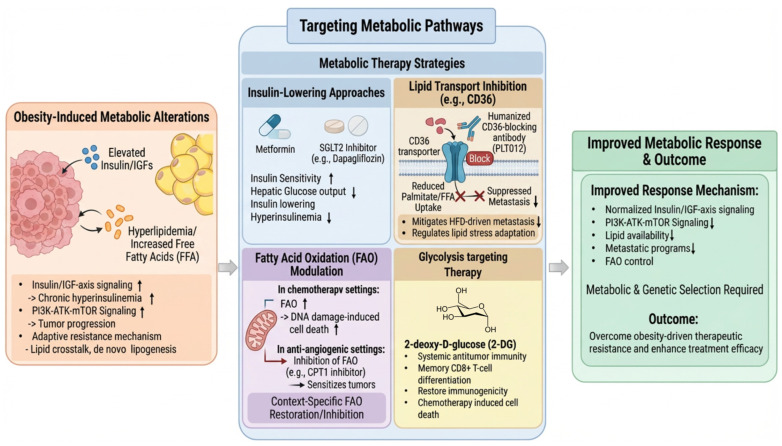
Therapeutic strategies targeting obesity-associated metabolic pathways to overcome cancer therapy resistance. Metabolic-targeted approaches aim to normalize tumor metabolic pathways and enhance treatment efficacy. Insulin-lowering interventions, including metformin and SGLT2 inhibitors, reduce hyperinsulinemia and attenuate insulin/IGF-driven signaling, thereby limiting tumor growth and survival. Lipid metabolism-targeting approaches, such as inhibition of CD36-mediated fatty acid transport, decrease lipid uptake and suppress lipid-driven metastasis and metabolic adaptation. In parallel, fatty acid oxidation (FAO) can be modulated in a context-dependent manner, either enhanced to promote chemotherapy-induced cell death or inhibited to disrupt metabolic flexibility under anti-angiogenic conditions. Additionally, glycolysis-targeting strategies, including 2-deoxy-D-glucose (2-DG), impair tumor energy metabolism and may enhance antitumor immune responses. Collectively, these approaches limit metabolic inputs that sustain tumor progression, constrain adaptive metabolic reprogramming, and improve therapeutic responsiveness.

Lastly, since obesity significantly alters lipid metabolism and glycolysis, targeting metabolic pathways presents a promising avenue for improving cancer therapy in obesity (Figure 5). A first metabolic target is attenuation of insulin/IGF-axis signaling, because chronic hyperinsulinemia in obesity can promote tumor progression directly and indirectly by sustaining growth and survival pathways downstream of PI3K–AKT–mTOR [108]. Metformin remains the best studied metabolic adjunct: it lowers glycemia primarily by suppressing hepatic glucose output and improving insulin sensitivity, actions mechanistically linked to effects on cellular energy state and mitochondrial function [109,110]. In diabetic breast cancer, a meta-analysis of 11 observational studies (5464 patients) reported that metformin exposure was associated with improved overall survival and a borderline or near-significant improvement in cancer-specific survival, supporting the hypothesis that insulin lowering can improve prognosis in insulin-resistant hosts [111]. In addition, a randomized phase II trial in EGFR-mutant advanced lung adenocarcinoma found that adding metformin to EGFR-TKI therapy significantly prolonged progression-free survival and overall survival compared with EGFR-TKIs alone, suggesting that systemic metabolic modulation can potentiate targeted therapy in selected biologic contexts, although the study was not designed as an obesity-only trial [112]. However, the largest modern randomized trial of metformin in non-diabetic early breast cancer (the MA.32 randomized clinical trial; 3649 patients) did not improve invasive disease-free survival overall, emphasizing that metabolic adjunct benefit is unlikely to be uniform across populations and may require metabolic and/or tumor-genetic selection [113]. Beyond metformin, sodium-glucose cotransporter 2 (SGLT2) inhibitors are emerging as stronger insulin-lowering tools in some contexts; in breast cancer mouse models spanning lean and diet-induced obesity, dapagliflozin reduced fasting hyperinsulinemia and slowed tumor growth, and in multiple models enhanced paclitaxel efficacy without obvious worsening of key chemotherapy-associated toxicities, supporting the concept that host insulin lowering can be leveraged as a precision metabolic adjuvant [114]; oncology evidence for SGLT2 inhibitors, however, remains confined to preclinical models and observational cohorts.

Targeting lipid metabolism represents another critical axis in adipose-rich tumor environments. Obesity increases lipid availability and promotes metabolic crosstalk between adipocytes and tumor cells, enabling adaptive resistance mechanisms through context-dependent remodeling of lipid uptake, fatty acid oxidation (FAO), survival signaling, and stress-response pathways. In anti-angiogenic therapy, treatment-induced hypoxia can drive lipid-dependent metabolic reprogramming, increasing fatty acid uptake and FAO [115]. In this context, inhibition of carnitine palmitoyltransferase 1 (CPT1), the rate-limiting step of FAO, sensitized tumors to anti-angiogenic therapy, supporting combined anti-angiogenesis plus anti-FAO strategies [115]. However, FAO modulation is not universally beneficial and exhibits strong context dependency. In chemotherapy settings, FAO can be required for effective tumor cell death. In a murine obesity model of melanoma, obesity impaired FAO induction through HIF-1α stabilization, resulting in chemoresistance, whereas pharmacologic restoration of FAO via PPARα activation improved treatment outcomes [82]. These findings argue against FAO inhibition as a universal strategy and instead support an obesity-aware, context-specific framework in which either inhibiting FAO or restoring FAO-mediated stress responses may be beneficial, depending on the underlying mechanism.

Beyond FAO, lipid metabolic reprogramming, including lipid uptake and de novo lipogenesis, also contributes to immune modulation and therapy resistance. Translationally, a humanized CD36-blocking antibody (PLT012) has been shown to reduce colorectal liver metastases and restore responsiveness to anti-PD-1 therapy in preclinical models, suggesting that targeting lipid uptake can reshape the metabolic landscape relevant to immunotherapy [116]. In parallel, targeting tumor lipogenesis also has important immunologic consequences. In ovarian cancer models, tumor-intrinsic fatty acid synthase (FASN) activity promoted lipid accumulation in tumor-infiltrating dendritic cells and impaired antitumor immunity, whereas pharmacologic FASN inhibition partially restored dendritic cell function and improved tumor control [117]. These findings support the concept that targeting lipid metabolism can potentiate immune-mediated therapeutic responses.

Finally, glycolysis-targeting strategies, although not yet extensively validated in obesity-specific contexts, provide mechanistic templates for rational combination approaches. For example, 2-deoxy-D-glucose (2-DG) has been shown to promote central memory CD8^+^ T-cell differentiation and enhance antitumor functionality [118,119,120]. In combination settings, particularly with chemotherapy, 2-DG can reprogram cancer cell death toward an immunogenic form, thereby enhancing chemotherapy-associated immunogenic cell death and supporting more durable T cell-mediated antitumor immune responses in preclinical models, suggesting that glycolysis modulation may enhance the immunogenicity of chemotherapy [118]. Clinically, however, 2-DG remains in early-phase development, with dose-limiting fatigue and cardiac toxicity that constrain its translational use.

Overall, these findings support metabolic reprogramming in obesity as a context-dependent therapeutic vulnerability, indicating that metabolism-targeted interventions may be most effective when aligned with the dominant metabolic dependencies of the tumor and its microenvironment.

### 5.4. Weight-Loss Interventions as a Therapeutic Adjuvant

Beyond pharmacologic strategies that target specific mechanisms within the tumor microenvironment, inflammation, or metabolism, addressing obesity itself through weight loss represents a conceptually attractive and increasingly evidence-based adjuvant approach. Three intervention classes are discussed: lifestyle modification, pharmacologic weight loss with GLP-1 receptor agonists, and bariatric surgery.

Lifestyle interventions (caloric restriction and exercise) have been shown to reverse obesity-induced TME alterations and normalize adipokine ratios in preclinical models, with re-sensitization to chemotherapy and anti-angiogenic therapy [121]. Clinical evidence from breast cancer survivors, including the LISA [122,123] and ENERGY [124] trials and ongoing BWEL trial [125], demonstrates improvements in circulating insulin, leptin-to-adiponectin ratio, and inflammatory biomarkers; disease-free survival endpoints from BWEL are awaited. Critically, the magnitude of weight loss achievable through lifestyle intervention alone is typically modest (5–8%), and durability beyond 12–18 months is limited.

GLP-1 receptor agonists (semaglutide, tirzepatide) achieve substantially greater and more durable weight loss (≥15%) and concurrently reduce systemic inflammation and insulin/IGF-1 signaling beyond what would be expected from weight loss alone [126]. Preclinical evidence in obese mouse models of breast and colorectal cancer shows reductions in tumor burden with GLP-1RA exposure [127]. However, cancer outcome data from large cardiovascular outcome trials such as SELECT remain immature, and ongoing FDA monitoring of a medullary thyroid carcinoma signal means that cancer-specific safety endpoints should be prospectively assessed [126,128]. Trials evaluating GLP-1RA as adjuvants to checkpoint inhibitors and chemotherapy are now beginning.

Bariatric surgery produces the greatest and most durable weight loss (≥25%) and is associated with substantial reductions in incident obesity-associated cancers (40–50% relative risk reduction) in large observational cohorts including the Swedish Obese Subjects study [129] and SPLENDID [130]. Preliminary data also suggest improved immunotherapy and chemotherapy outcomes after surgical weight loss in patients with obesity-associated malignancies. Caveats include selection bias, altered drug due to changes in nutrient absorption and gastric anatomy, and the absence of randomized controlled trials testing cancer endpoints as primary outcomes.

Critical appraisal and unresolved questions. Randomized trials of weight-loss interventions as cancer therapy adjuncts (e.g., BWEL [125] and emerging GLP-1RA cancer adjuvant trials in breast and endometrial cancer [131]) are still maturing. The optimal timing (pre-diagnosis vs. peri-treatment), magnitude (≥5% vs. ≥10% body weight), and durability of weight loss required to overcome therapy resistance remain undefined. Furthermore, the relative contribution of weight loss *per se* versus pleiotropic effects of pharmacologic agents (e.g., GLP-1RA effects on inflammation, or surgically induced changes in gut microbiota and bile acid signaling) has not been clearly delineated [131,132]. Until these questions are resolved, weight-loss interventions should be considered a promising but still investigational adjuvant strategy in obesity-associated cancers.

## 6. Conclusions

Obesity accelerates cancer progression and reduces responsiveness to major cancer treatment modalities, including chemotherapy, immunotherapy, and radiotherapy, through changes in the TME, inflammation, and metabolic reprogramming. Further research is needed to clarify this relationship and to develop targeted, personalized therapeutic strategies for patients with cancer who have obesity.

Several key conceptual advances emerge from the work summarized in this review. First, the obesity-conditioned tumor microenvironment constitutes a distinct biological state, not merely a quantitative extension of the lean-host TME, shaped by adipose tissue-driven cytokines, adipokines, hormonal shifts (notably aromatase-mediated estrogen synthesis), and persistent metabolic stress. Second, metabolic reprogramming in obesity is context-dependent: pathways such as fatty acid oxidation can act as drivers of resistance in anti-angiogenic settings but as effectors of cell death in chemotherapy settings, arguing against universal metabolic inhibition strategies. Third, the obesity paradox in immunotherapy reframes obesity as a modifiable, rather than uniformly detrimental, determinant of immune checkpoint response, underscoring the need for context-aware interpretation of inflammation.

Clinical implications: Routinely incorporating BMI, body composition (e.g., visceral adiposity, sarcopenia), and host metabolic biomarkers, including circulating insulin, the leptin-to-adiponectin ratio, and inflammatory indices such as IL-6, IL-8, and the systemic immune-inflammation index, into oncology decision-making would represent a substantial advance over current practice. The therapy modalities most affected by obesity—chemotherapy, immunotherapy, and radiotherapy—should be the primary focus of obesity-aware treatment optimization.

Current limitations: Much of the mechanistic evidence remains based on diet-induced obese mouse models that do not fully recapitulate human obesity. The term “obesity” itself includes substantial heterogeneity (metabolically healthy vs. unhealthy obesity, central vs. peripheral adiposity, sarcopenic obesity), and patients with severe obesity are underrepresented in pivotal oncology trials. Obesity-stratified randomized clinical trials remain the exception rather than the norm. This narrative review, like the underlying literature, is also subject to English-only inclusion and selective citation of key studies.

Future directions: We propose four priorities for the next phase of research: (i) obesity-aware clinical trial design, with prospective BMI/body-composition stratification, pre-specified obesity subgroup analyses, and inclusion of metabolic endpoints; (ii) biomarker development, including standardization of circulating adipokines and inflammatory indices and validation of imaging-based body-composition measures as predictive and pharmacodynamic biomarkers; (iii) patient stratification strategies that integrate host metabolic phenotyping with tumor metabolic profiling to guide selection of metabolic adjuvants (metformin, SGLT2 inhibitors, and GLP-1RA) and weight-loss interventions; and (iv) mechanism-aligned combination trials evaluating weight-loss interventions in combination with checkpoint inhibitors, anti-angiogenic therapy, or chemotherapy in obesity-enriched cohorts. Advancing these priorities will require a concerted, multidisciplinary effort integrating clinical, translational, and basic research to deliver effective and personalized therapies for patients with cancer who have obesity.

## Data Availability

No new data were created or analyzed in this study.

## Abbreviations

The following abbreviations are used in this manuscript:

TME	Tumor microenvironment
HIF	Hypoxia-inducible factor
EMT	Epithelial–mesenchymal transition
ECM	Extracellular matrix
MDSC	Myeloid-derived suppressor cell
TNBC	Triple-negative breast cancer
PDAC	Pancreatic ductal adenocarcinoma
IGF-1	Insulin-like growth factor 1
iNOS	Inducible nitric oxide synthase
HAP	Hypoxia-activated prodrug
HBO	Hyperbaric oxygen
PD-1	Programmed cell death protein 1
FAS	Fatty acid synthase
FAO	Fatty acid oxidation
SGLT2	Sodium-glucose cotransporter 2
GLP-1RA	GLP-1 receptor agonist
